# Genetic Linkage Map Construction and QTL Analysis of Two Interspecific Reproductive Isolation Traits in Sponge Gourd

**DOI:** 10.3389/fpls.2016.00980

**Published:** 2016-07-07

**Authors:** Haibin Wu, Xiaoli He, Hao Gong, Shaobo Luo, Mingzhu Li, Junqiu Chen, Changyuan Zhang, Ting Yu, Wangping Huang, Jianning Luo

**Affiliations:** ^1^Vegetable Research Institute, Guangdong Academy of Agricultural ScienceGuangzhou, China; ^2^Guangdong Provincial Key Lab for New Technology Research on VegetablesGuangzhou, China; ^3^Agro-biological Gene Research Center, Guangdong Academy of Agricultural SciencesGuangzhou, China

**Keywords:** sponge gourd, EST-SSR marker, genetic linkage map, QTL mapping, flowering time, pollen fertility

## Abstract

The hybrids between *Luffa acutangula* (L.) Roxb. and *L.cylindrica* (L.) Roem. have strong heterosis effects. However, some reproductive isolation traits hindered their normal hybridization and fructification, which was mainly caused by the flowering time and hybrid pollen sterility. In order to study the genetic basis of two interspecific reproductive isolation traits, we constructed a genetic linkage map using an F2 population derived from a cross between S1174 [*L. acutangula* (L.) Roxb.] and 93075 [*L. cylindrica* (L.) Roem.]. The map spans 1436.12 CentiMorgans (cM), with an average of 8.11 cM among markers, and consists of 177 EST-SSR markers distributed in 14 linkage groups (LG) with an average of 102.58 cM per LG. Meanwhile, we conducted colinearity analysis between the sequences of EST-SSR markers and the genomic sequences of cucumber, melon and watermelon. On the basis of genetic linkage map, we conducted QTL mapping of two reproductive isolation traits in sponge gourd, which were the flowering time and hybrid male sterility. Two putative QTLs associated with flowering time (FT) were both detected on LG 1. The accumulated contribution of these two QTLs explained 38.07% of the total phenotypic variance (PV), and each QTL explained 15.36 and 22.71% of the PV respectively. Four QTLs for pollen fertility (PF) were identified on LG 1 (*qPF1.1* and *qPF1.2*), LG 3 (*qPF3*) and LG 7 (*qPF7*), respectively. The percentage of PF explained by these QTLs varied from 2.91 to 16.79%, and all together the four QTLs accounted for 39.98% of the total PV. Our newly developed EST-SSR markers and linkage map are very useful for gene mapping, comparative genomics and molecular marker-assisted breeding. These QTLs for interspecific reproductive isolation will also contribute to the cloning of genes relating to interspecific reproductive isolation and the utilization of interspecific heterosis in sponge gourd in further studies.

## Introduction

Sponge gourd (2n = 26), also called luffa, is a cross-pollinated diploid species from the cucurbitaceous family, which is mainly cultivated in tropical and subtropical regions, such as China, Malaysia, India, Thailand, Central America, and Africa (Oboh and Aluyor, 2009; Rabei et al., 2013; Wu et al., 2014). Nine varied species of sponge gourd are distributed around the world, of which only *Luffa acutangula* (L.) Roxb. and L.cylindrica (L.) Roem. are cultivated (Prakash et al., 2013; Rabei et al., 2013; Wu et al., 2014). They obviously differ from each other in that *L. acutangula* fruit possesses deep grooves and thus is also called ridged luffa, while the surface of *L. cylindrica* is smooth, due to which it is also called smooth luffa.

The fruit of sponge gourd is rich in nutrition. The immature fruit can be enjoyed as a vegetable, and the fully-grown sponge gourd can be used as household cleaning products and industrial raw materials. In particular, sponge gourd contains multiple bioactivators, such as alkaloids, flavonoids, sterols, glycosides and glycoprotein, and possesses anti-inflammation, anti-fungal, anti-bacterial, anti-myocardial ischemia, sedative and analgesic activities (Ng et al., 1992a,b; Partap et al., 2012; Wu et al., 2014). Meanwhile, ribosome-inactivating protein in the seeds of sponge gourd contains anti-HIV activity (Ng et al., 1992b, 2011).

A genetic linkage map is vital for mapping genes/QTLs controlling desirable agronomic traits, comparative genomic research, and marker assisted selection. The saturated genetic linkage maps (< 1 cM between markers) have been constructed in cucumber, melon and watermelon, while similar study of sponge gourd is rarely found (Ren et al., 2009, 2012; Diaz et al., 2011). By making use of sequence related amplified polymorphism (SRAP) markers, Cui et al. (2015) constructed a genetic map consisting of 258 loci on 24 linkage groups, of which the overall length was 822.86 cM and the mean interval between markers was 3.49 cM, becoming the first genetic linkage map of sponge gourd in the world. However, due to the fact that the SRAP is a kind of random primer amplified marker, its accuracy and maneuverability are not quite satisfying. The SSR markers show huge advantage in the construction of molecular genetic maps since they are more accurate, operative and plentiful. Previously, we identified 8523 pairs of EST-SSR markers in sponge gourd through transcriptome sequencing for the first time, of which 641 pairs of markers were verified, and then employed 50 pairs of them to study the diversity of 60 sponge gourd accessions (Wu et al., 2014), which can contribute to the construction of sponge gourd genetic linkage map.

Heterosis or hybrid vigor is a common natural phenomenon in the biological world, and reproductive isolation happens when two parents are distantly related in genetic relationship. Reproductive isolation serves as the indicator of speciation, and is also a mechanism to maintain the purity of species (Orr and Presgraves, 2000; Long et al., 2008; Hinchliffe et al., 2011). Dobzhansky-Muller model argued that hybrid incompatibility was caused by the accumulative negative interaction of two or more loci. As for animals, several genes resulting in hybrid incompatibility have been identified in Drosophila and mice (Morán and Fontdevila, 2014; Civetta and Gaudreau, 2015; Davies et al., 2016). As for plants, correlated studies in rice have been conducted in depth and a few genes causing hybrid sterility have been cloned (Long et al., 2008; Yang et al., 2012; Chen and Liu, 2014). In the case of Sponge gourd, the hybrids with crossed between *L. acutangula* and *L. cylindrica* have strong heterosis effects. However, some reproductive isolation traits hindered their normal hybridization and fructification. At present the research about reproductive isolation in sponge gourd can be scarcely found, and our studies showed that the reproductive isolation between *L. acutangula* and *L. cylindrica* mainly consisted of two aspects. Firstly, there was almost a 12-h gap in their flowering time. During the day, the former was usually from 5 to 7 p.m., while the latter often blossomed from 4 am to 6 am, leading to the difficulty of natural hybridization between them. Secondly, they showed hybrid male sterility. After the artificial hybridization between them, hybrid F1 was in half sterile condition.

On the basis of EST-SSR markers previously developed by transcriptome sequencing, this research continued to develop 405 pairs of EST-SSR markers, eventually obtaining 1046 pairs of EST-SSR markers in sponge gourd. Furthermore, we constructed a genetic linkage map using an F2 population derived from a cross between S1174 (*L. acutangula*) and 93075 (*L. cylindrica*). Then we also conducted QTL mapping of two traits of reproductive isolation in sponge gourd, which were the flowering time and hybrid male sterility. Our studies are supposed to contribute to the following cloning of genes relating to interspecific reproductive isolation, the utilization of the heterosis of interspecific, and the development of molecular marker assisted breeding in sponge gourd.

## Materials and methods

### Plant materials

There are two advanced inbred lines, S1174 and P93075, which belonged to *L. acutangula* (L.) Roxb. and *L. cylindrical* (L.) Roem. respectively and used as experimental materials. The mapping population consisting of 186 F2 individuals was generated by cross of S1174 × P93075.

### Trait measurements and statistical analysis

Two traits of reproductive isolation relating to flowering time and hybrid pollen fertilities were investigated. During the bloom stages of the mapping population, the flowering time of each plant was investigated from 4:00 p.m. to 7:00 a.m. for three successive days, and took the average value as the flowering time of every plant. Considering the convenience of statistical analysis, we need to transform the recorded time data to decimal time measured in hour, and assigned 0 to the plants with flowering time at 0:00, negative value to those with flowering time before 0:00, and positive value to those after 0:00. For example, if the flowering time was at 1:30 a.m., it was transformed to 1.5.

During the full-bloom stages of the mapping population, three male flowers were fetched from each plant, immersed in FAA fixative (3.7% v/v formaldehyde, 50% ethanol, 5% acetic acid) and brought back to the lab. By observing the pollen fertility through a microscope, we chose one field of vision with pollens distributed evenly for each male flower, and counted the number of fertile pollens and sterile pollens in each field of vision. Three male flowers with three fields of vision were analyzed and the fertile pollen rate in each field was calculated, taking their average value as the fertile pollen rate of single plant.

Statistical analysis on the phonotypic traits of parental groups and mapping population were carried out by adopting SPSS software. After computing the frequency distribution parameters and normal distribution parameters, their diagrams were drawn with SIGEMA-PLOT.

### DNA extraction and molecular marker analysis

Genomic DNA was isolated from sponge gourd young leaves using the cetyltrimethylammonium bromide (CTAB) method (Saghai-Maroof et al., 1984). PCR amplifications were performed in a 20 μL reaction volume containing 100 ng genomic DNA, 1 × PCR buffer, 2 mM MgCl_2_, 2.5 mM dNTPs, 4 μM of each primer, and 1 U Taq polymerase.

Amplification was performed on an Applied Biosystems 9700 thermocycler following the touchdown protocol: using 94°C for 5 min; followed by 11 cycles of 94°C for 30 s, 65–55°C for 30 s decreasing 1°C per cycle, and 72°C for 1 min; followed by 30 cycles of 94°C for 40 s, 55°C for 30 s, 72°C for 1 min; and a final extension at 72°C for 10 min. PCR products were performed on an 8% polyacrylamide gel. Gels were stained with silver nitrate as previously described (Bassam et al., 1991).

### Linkage map construction and QTL analyses

JoinMap version 4.0 software (Van Ooijen, 2006) was used to construct the linkage maps at LOD scores ≥4.0. The Kosambi mapping function was used to calculate the genetic distance between markers. The genetic map was drawn with MapDraw (Liu and Meng, 2003).

Candidate QTL regions were identified by using Windows QTL Cartographer ver. 2.5 (Wang et al., 2007). Composite interval mapping (CIM) procedure was performed using the Model 6, with the window size set at 10 cM and a walking speed of 1 cM. The genome-wide LOD score threshold (*a* = 0.05) for declaring the presence of QTLs was determined using the permutation test (1000 replications). Based on the permutation results, the LOD score threshold was set at 3.0 for the trait to declare the presence of a significant QTL. The additive effect and percentage of phenotypic variance explained by each QTL were estimated at the peak LOD score.

### Genome comparative mapping

To detect cross-species synteny, each amplicon or unigene of sponge gourd was BLASTN searched against the genome sequences of cucumber (Huang et al., 2009), melon (Garcia-Mas et al., 2012), and watermelon (Guo et al., 2013) and the sequences were considered orthologous if sharing ≥80% sequence identity with an *e* ≤ 1e-5. In cases where multiple hits occurred, only the best hits were used. The software Circos (Krzywinski et al., 2009) was employed to visualize the genome syntenic relationships.

## Results

### Mark development

By means of transcriptome sequencing of sponge gourd in the previous experiment, we developed 8523 high-quality SSR primer pairs, and 641 primer pairs were synthesized and verified (Wu et al., 2014). In current experiment 405 markers were synthesized, and the polymorphisms and successful amplification of them were verified in S1174, 93075 and their hybrid F1 (Supplementary Material 1). Of these primers, 80, 81, 60, 62, 62, and 60 were for di-, tri-, tetra-, pena-, hexa-nucleotide repeats, and compound formation repeats, respectively (Table 1). A total of 325 (80.25%) exhibited successful amplification, of which 216 (66.46%) revealed polymorphism between S1174 and 93075. Polymorphisms could be observed for 41 di-, 33 tri-, 28 tetra-, 39 penta-, 42 hexa-nucleotide repeats and 33 compound formation repeats. Among the 216 polymorphic primer pairs, 101 (46.76%) were co-dominant and 115 (53.24%) were dominant.

**Table 1:** **Characteristics of synthesized EST–SSRs and efficiency of marker development**.

**Motif**	**No. of EST-SSRs**	**No. of amplified EST-SSRs^a^ (%)**	**No. of polymorphic EST-SSRs^b^ (%)**	**No. of co-dominant EST-SSRs^c^ (%)**	**No. of dominant EST-SSRs^d^ (%)**
Di-nucleotide	80	70 (87.50%)	41(58.57%)	17(41.46%)	24(58.54%)
Tri-nucleotide	81	63 (77.78%)	33(52.38%)	14(42.42%)	19(57.58%)
Tetra-nucleotide	60	48 (80.00%)	28(58.33%)	14(50.00%)	14(50.00%)
Penta-nucleotide	62	50 (80.65%)	39(78.00%)	21(53.85%)	18(46.15%)
Hexa-nucleotide	62	51 (82.26%)	42(82.35%)	23(54.76%)	19(45.24%)
Compound	60	43 (71.67%)	33(76.74%)	12(36.36%)	21(63.64%)
Total	405	325 (80.25%)	216(66.46%)	101(46.76%)	115(53.24%)

a *Percentage of successfully amplified EST–SSRs per synthesized primer pair*.

b *Percentage of polymorphic markers per amplified primer pair*.

c *Percentage of co-dominant markers per polymorphic primer pair*.

d *Percentage of dominant markers per polymorphic primer pair*.

A total of 1046 EST-SSR markers from this experiment and the previous experiment (Wu et al., 2014) were used to screen the parental lines S1174 and 93075 for polymorphic markers. Of the 1046 EST-SSR primers, 417 (39.87%) generated clear and scorable polymorphic bands between the parental lines. Among the 417 polymorphic primer pairs, 227 (62.69%) were co-dominant and 190 (37.31%) were dominant. The 417 EST-SSR polymorphic markers were used to construct the linkage map with the F2 population of S1174 × P93075, in which only 177 markers were left after deleting those with blurry bands or hard to be linked to map.

### Genetic linkage map construction

**Figure 1:**
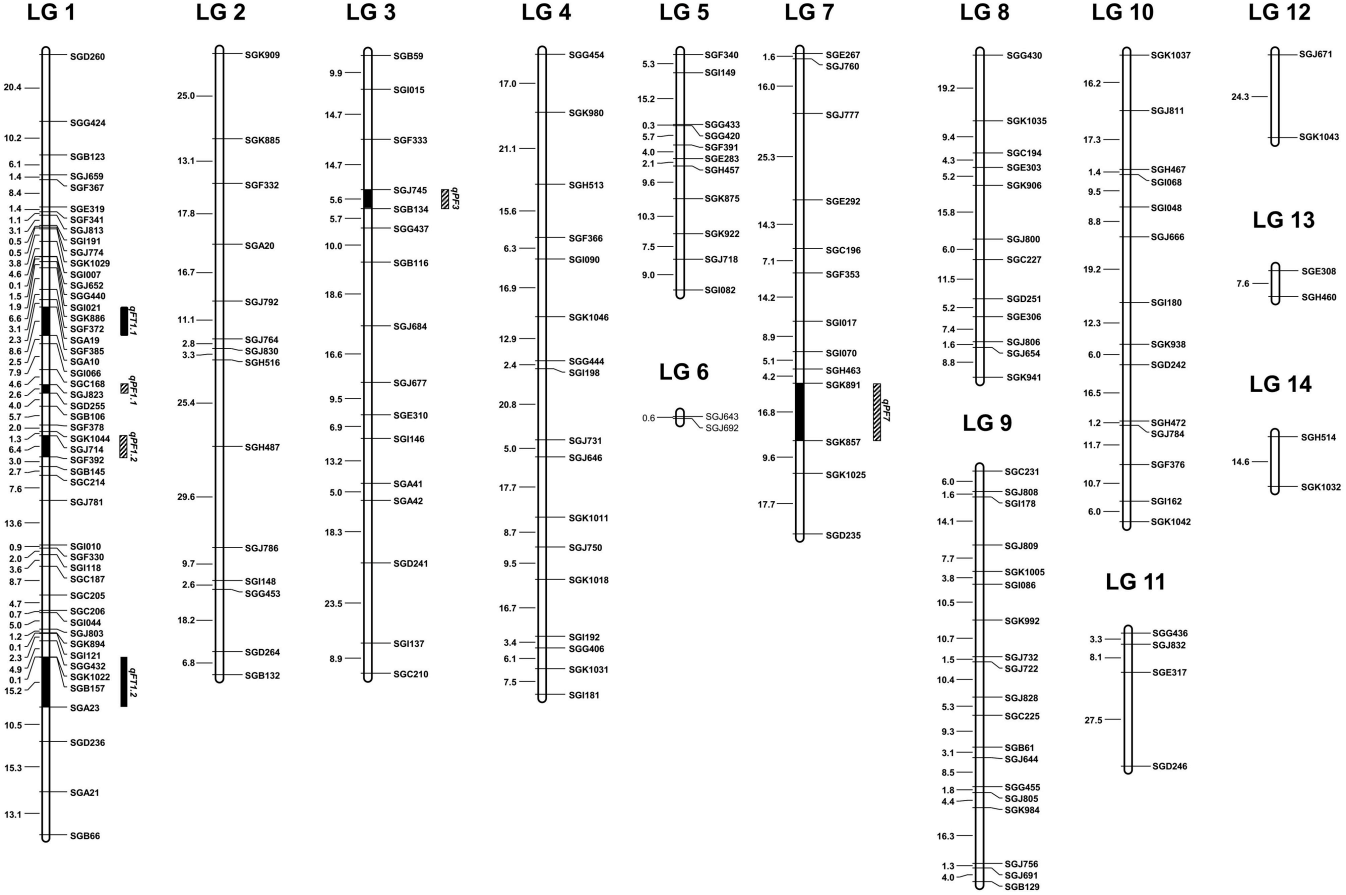
**The 14 linkage groups (LG 1-LG 14) of sponge gourd base on the F2 population derived from the cross S1174 × P93075**. The EST-SSR markers were shown on the right of the LG, and distances among markers were indicated in cM on the left. The QTLs for flowering time (*qFT*) were mapped to LG 1. The QTLs for pollen fertility (*qPF*) were mapped to LG 1, LG 3 and LG 7.

The 177 polymorphic EST-SSR markers between S1174 and P93075 were mapped on 14 linkage groups, of which 164 were codominant and 13 dominant, and covered a genetic length of 1436.12 CentiMorgans (cM) (Table 2, Figure 1). Because of the inter-specific hybridization, there are 48 pairs of EST-SSR markers deviating 1:2:1 (codominant markers) or 3:1 (dominant markers) (α = 0.05) in the 177 markers. The map length of the 14 linkage groups ranged from 0.62 cM (LG) to 237.61 cM (LG) with an average of 102.58 cM per LG. The density of markers ranged from 0.62 cM (LG) to 14.01 cM (LG), with an average of 8.11 cM per LG.

**Table 2:** **Distribution of molecular markers among 14 linkage group (LGs) established on a genetic map using an F2 population derived from the cross S1174 × P93075**.

**Linkage groups**	**Map length (cM)**	**No. of markers**	**Marker density (cM/marker)**
LG 1	237.61	49	4.85
LG 2	182.07	14	14.01
LG 3	181.27	16	12.081
LG 4	187.61	17	11.73
LG 5	69.14	11	6.91
LG 6	0.62	2	0.62
LG 7	140.83	13	11.73
LG 8	94.44	12	8.59
LG 9	120.24	19	6.68
LG 10	136.89	14	9.78
LG 11	38.89	4	9.72
LG 12	24.3	2	24.3
LG 13	7.61	2	7.61
LG 14	14.6	2	14.6
Total	1436.12	177	8.11

### Cross-species synteny

**Figure 2:**
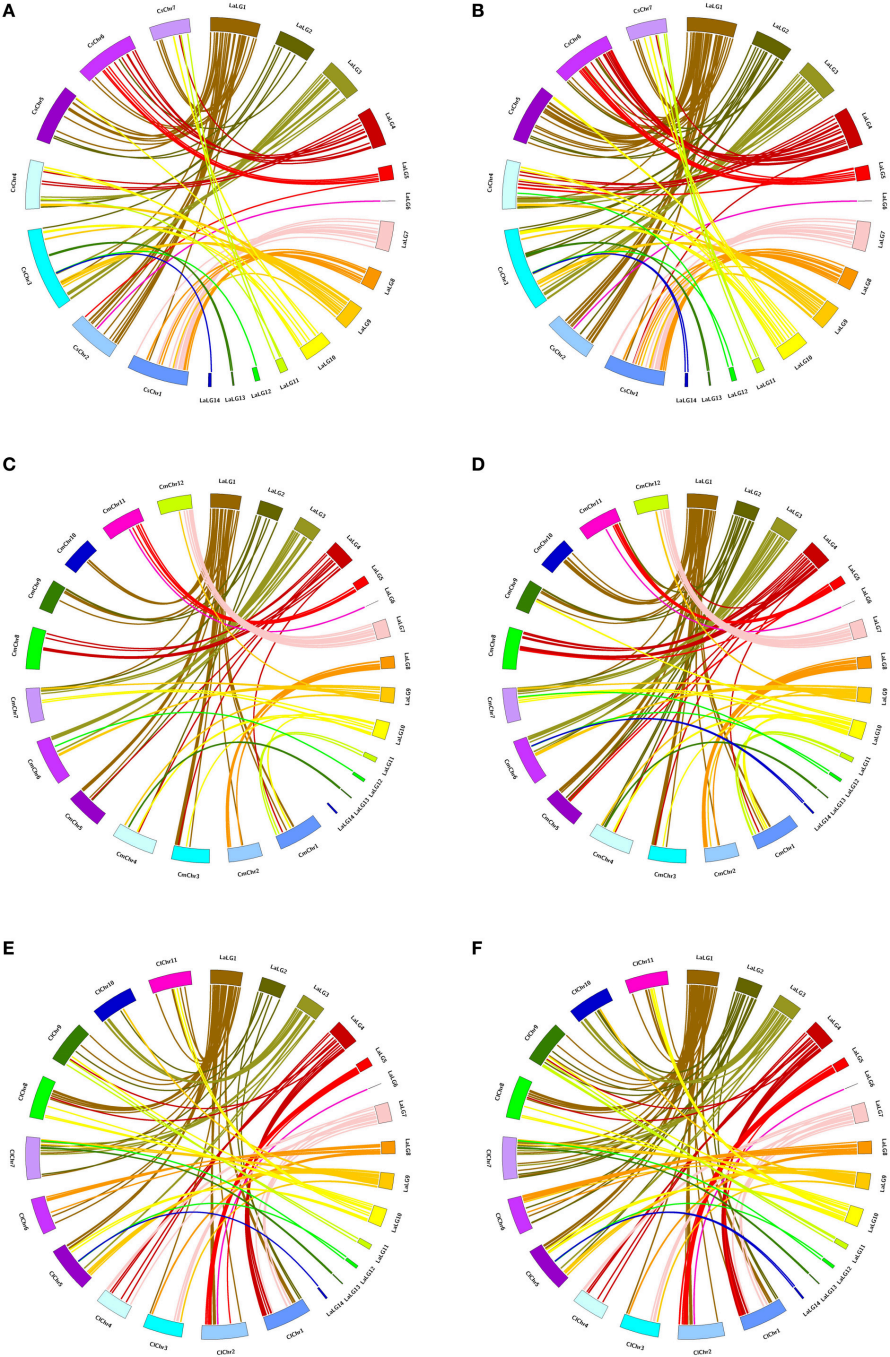
**Circos illustration of the colinearity analysis between the sequences of sponge gourd EST-SSR markers and the genomic sequences of cucumber, melon and watermelon. (A)** The PCR-amplified target fragments of sponge gourd EST-SSR markers–cucumber genome. **(B)** The Unigenes where sponge gourd EST-SSR markers located—cucumber genome. **(C)** The PCR-amplified target fragments of sponge gourd EST-SSR markers—melon genome. **(D)** The Unigenes where sponge gourd EST-SSR markers located—melon genome. **(E)** The PCR-amplified target fragments of sponge gourd EST-SSR markers—watermelon genome. **(F)** The Unigenes where sponge gourd EST-SSR markers located—watermelon genome. The sponge gourd linkage groups are denoted as LaLGs and the pseudomolecules of cucumber, watermelon and melon are represented as CsChrs, ClChrs, and CmChrs, respectively.

Of 177 pairs of EST-SSR molecular markers in genetic linkage map, the PCR-amplified target fragments and Unigenes where markers located were used to conduct syntenic analysis with the genomic sequences of cucumber, melon and watermelon, respectively. Among the amplified target fragments of 177 pairs of markers, 119, 111, and 135 have orthoglogs in genomes of cucumber, melon and watermelon, respectively (Figure 2). Among the Unigenes where 177 pairs of markers located, 169,167 and 172 have orthoglogs in genomes of cucumber, melon and watermelon, respectively (Figure 2). Each sponge gourd LG matched one to eight chromosomes of cucumber and melon, and up to nine chromosomes of watermelon (Supplementary Materials 2–4).

### Phenotypic data

**Figure 3:**
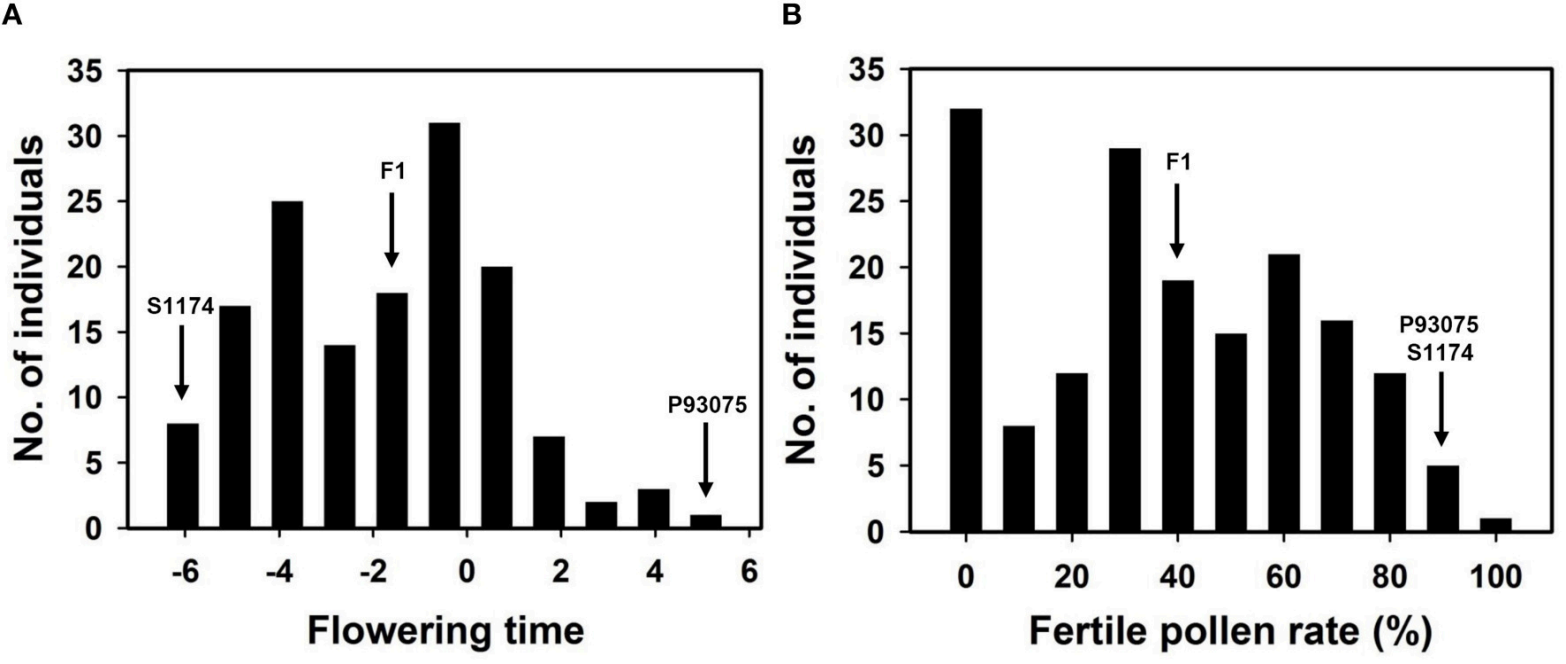
**Distribution of flowering time (A) and pollen fertility (B) in F2 population derived from the cross S1174 × P93075**.

During the blossoming period of sponge gourd, the flowering time of *L. acutangula* was usually from 5:00 to 7:00 p.m., while *L. cylindrica* normally opened flowers from 4:00 to 6:00 a.m., and there were about 12-h difference in their flowering times. The statistic data provided in this research was gained during April 21–23 in 2012 in Guangzhou: S1174 (*L. acutangula*) bloomed at 5:32 p.m. (the number became -6.47), P93075 (*L. cylindrica*) bloomed at 4:57 (the number became 4.95) and F1 bloomed at 10:30 p.m. (the number became -1.5), which was between the flowering time of male parent and female parent and closer to the latter. As for F2 population, its flowering time ranged from 5:57 p.m. to 5:05 a.m. (number became -6.05–5.08), and the average time would be 10:43:48 p.m. (number became -1.27; Table 3, Figure 3).

**Table 3:** **Flowering time (FT) and pollen fertility (PF) of parental lines S1174, P93075, F1 hybrids, and F2 population derived from the cross S1174 × P93075**.

**Trait**	**Parental lines^a^**		**F1**	**F2 population**				
	**S1174**	**P93075**		**Min**	**Max**	**Mean**	**Skewness**	**Kurtosis**
FT (h)^b^	−6.5 (17:30)	5.03 (5:02)	−1.45 (22:34)	−6.05 (17:57)	5.08 (5:05)	−1.27 ± 2.41	0.17	−0.39
PF (%)	94.31	95.54	44.71	0	100	42.83 ± 28.35	−0.07	−1.05

a *The mean value of the results of the three measurements*.

b *Decimal time measured in hour which is transformed in the way used in “Materials and Methods”; standard time in brackets*.

**Figure 4:**
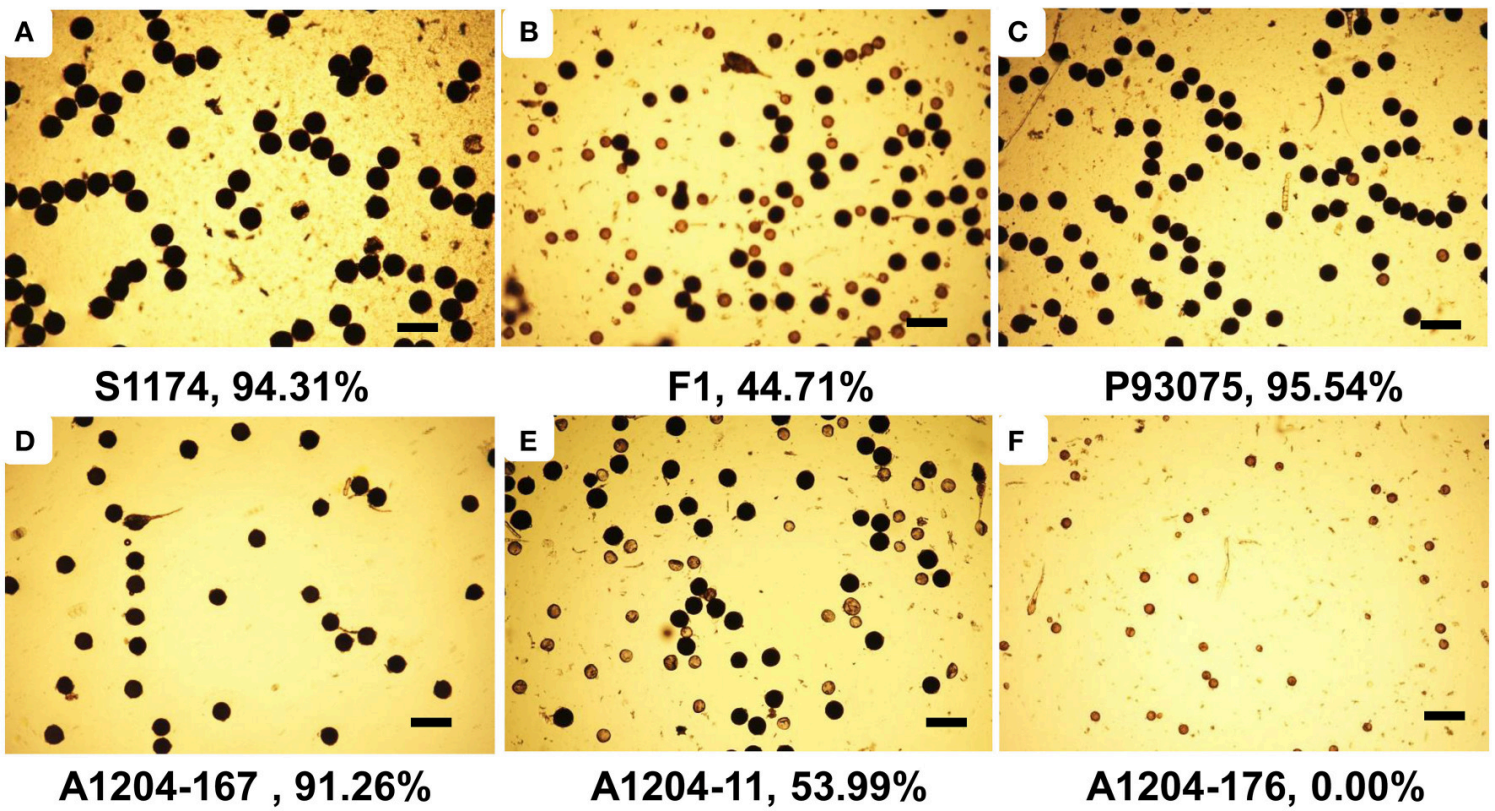
**Pollen phenotypes of S1174 (A), P93075 (C), F1 (B), and F2 plants (D–F)**. Three male flowers with three fields of vision were analyzed and the fertile pollen rate in each field was calculated, taking their average value as the fertile pollen rate of single plant. Bars = 200 μm.

Under normal cultivation conditions (exclude all kinds of harsh environments, such as drought, flood, extreme high or low temperature), the pollen of the parent of S1174 and the parent of P93075 was complete fertile (the fertility rate was above 94%). The pollen of F1 plants was semi-sterile (44.71%), while the pollen of F2 individuals present continuous variation with pollen fertility ranging from complete fertile (100%) to complete sterile (pollen free), and the average pollen fertility rate was 43% (Table 3, Figure 4).

### QTL analysis

The QTLs for flowering time and pollen fertility of sponge gourd are summarized in Table 4. Two putative QTLs associated with flowering time (FT) were both detected on LG 1. The accumulated contribution of these QTLs explained 38.07% of the total phenotypic variance (PV), and individual QTL explained 15.36 and 22.71% of the PV. The QTLs *qFT1.1* showed additive and the QTL *qFT1.2* showed overdominance. Moreover, these QTLs increased FT by the presence of P93075 alleles. A major QTL *qFT1.1* was mapped on LG 1 flanked by SGF385 and SGA10, which accounted for 22.71% of the PV (Table 4, Figure 1). The marker SGF385 distributed at one side of *qFT1.1* with a distance of 3.02 cM. On the other side of *qFT1.1*, the marker SGA10 was located at a distance of 5.54 cM. The other major QTLs *qFT1.2* was also mapped on LG 1, which accounted for 15.36% of the PV (Table 4, Figure 1).The marker SGB157 was distributed at a distance of 8.00 cM to *qFT1.3* and the marker SGA23 on the other side of *qFT1.3* was located with a distance of 7.19 cM.

**Table 4:** **Summary of QTLs for flowering time (FT) and pollen fertility (PF) using composite interval mapping in F2 population derived from the cross S1174 × P93075**.

**Trait**	**QTL^a^**	**Linkage group**	**Marker Interval^b^**	**Position (cM)**	**LOD Score**	**Additive effect^c^**	**Dominace effect^d^**	**R^2^^e^**	**D/A^f^**	**GA^g^**
FT	*qFT1.1*	1	SGF385-SGA10	79.90	6.88	−1.73	0.12	22.71	−0.07	A
	*qFT1.2*	1	SGB157-SGA23	191.50	3.40	−0.81	1.47	15.36	−1.81	OD
PF	*qPF1.1*	1	SGJ823-SGD255	101.50	5.01	0.99	−20.85	9.12	−21.06	OD
	*qPF1.2*	1	SGJ714-SGF392	117.10	3.85	−11.48	−17.91	2.91	1.56	OD
	*qPF3*	3	SGJ745-SGB134	42.40	3.01	−6.75	15.83	11.16	−2.34	OD
	*qPF7*	7	SGK891-SGK857	99.70	3.65	10.18	−13.04	16.79	−1.28	OD

a *Individual QTLs are shown with the italic abbreviation of the trait and the linkage group number*.

b *The marker interval to the putative QTL is shown in bold in Figure 1*.

c *Positive or negative value indicates that the allele from S1174 or P93075 increases the phenotypic value, respectively*.

d *Positive or negative value indicates the effect increasing or decreasing trait value over the population mean*.

e *Percentage of the total phenotypic variation explained by the QTL*.

f *D/A Dominance/Additive*.

g *GA gene action modes classified as A additive (|d/a| = 0–0.2), PD partial dominance (|d/a| = 0.21–0.80), D dominance (|d/a| = 0.81–1.20), and OD overdominance (|d/a| > 1.2)*.

Four QTLs for pollen fertility (PF) were identified on LG 1 (*qPF1.1* and *qPF1.2*), LG 3 (*qPF3*), and LG 7 (*qPF7*), respectively. The percentage of PF explained by these QTLs varied from 2.91 to 16.79%, and all together the four QTLs accounted for 39.98% of the total phenotypic variance. These QTLs all showed overdominance. The PF was increased by the presence of S1174 alleles at *qPF1.1* and *qPF7* and by the presence of P93075 alleles at *qPF1.2* and *qPF3*, among which the interpretable phenotypic variance of two QTLs (*qPF3* and *qPF7*) was greater than 10%. The *qPF3* was mapped on LG 3 flanked by SGJ745 and SGB134 which explained 11.16% of the PV (Table 4, Figure 1). The marker SGJ745 was distributed at one side of *qPF3* with a distance of 3.03 cM and the marker SGB134 on the other side of *qPF3* was located at a distance of 2.56 cM. The *qPF7* was identified on LG 7 flanked by SGK891 and SGK857 which explained 16.79% of the PV (Table 4, Figure 1). The marker SGK891 was located at a distance of 3.01 cM to *qFT 7* and the marker SGK857 on the other side of *qPF 7* was distributed with a distance of 13.83 cM.

## Discussion

It's necessary for genetic linkage map construction and QTL mapping to keep sufficient molecular markers. EST-SSR markers are easy to use, stable to amplify, high in specificity, and regarded as functional markers since they are derived from transcripts. It will effectively eliminate the interference from invalid markers and improve the efficiency of gene mapping. Wu et al. (2014) synthesized and verified 641 EST-SSR markers in sponge gourd, and furthermore, 405 EST-SSR markers were synthesized and verified in this research, getting 1046 verified markers in sponge gourd, which can meet basically the requirement of molecular genetic study of sponge gourd. In addition, this research also witnessed high rate of successful amplification (80.25%) and high level of polymorphism between *L. acutangula* and *L. cylindrica*.

Accurate genetic linkage map serves as the basis for QTL analysis. However, the research work concerning genetic linkage map just got started in sponge gourd. Recently, Cui et al. (2015) reported a sponge gourd linkage map consisted of 258 SRAP loci covering 822.86 cM and distributed on 24 linkage groups with an average distance of 3.49 cM between makers. This map can be regarded as the first sponge gourd genetic linkage map in the world, which will contribute to the molecular genetic study of sponge gourd. But there are a few disadvantages in this map. First of all, SRAP (sequence related amplified polymorphism) markers selected in this map were a kind of non-specific amplification molecular marker. For the first five cycles, the annealing temperature of SRAPs was set at 35°C and introduced with plenty of non-specific amplification, resulting in lower accuracy and reproducibility. Therefore, if the entire genetic linkage map was constructed with SRAP markers, the low accuracy and generality of the map would make it difficult to make comparison between various results, affecting the following study of genes/QTLs mapping. Secondly, there are 13 pairs of chromosomes in sponge gourd, while this map possesses 24 linkage groups, indicating the poor linkage among markers, for which small linkage groups can hardly form bigger linkage groups. In this study, a comprehensive genetic linkage map was constructed by using 177 EST-SSR markers. The map had a total coverage of 1436.12 cM and distributed on 14 linkage groups. Because of the inter-specific hybridization, the segregation distortion of molecular markers of sponge gourd was quite serious, with 48 pairs of markers deviating 1:2:1 (codominant markers) or 3:1 (dominant markers) (α = 0.05). Since the segregation distortion of alleles was closely related to hybrids sterility, molecular markers with segregation distortion weren't discarded during the construction of linkage map. Compared with the study of Cui et al. (2015), this map only selected specific molecular markers, and the map was almost twice as long as that reported by Cui et al. (2015), with more concentrated markers, less linkage groups and closer chromosome number to sponge gourd (2n = 26). To our knowledge, this map is the first genetic linkage map comprising specific molecular markers all over the world.

This research constructed a genetic linkage map consists of 177 EST-SSR markers distributed unevenly on linkage groups. Each linkage group only get a dozen markers or even several markers except LG1 having 49 markers. The reason for causing this situation could be the uneven gene distribution on chromosome (Lou et al., 2013; Kodama et al., 2014). There are more molecular markers in areas with more gene expression, while the less markers in heterochromatin areas with less gene expression. For genetic map alone, it may be not quite complete if using EST-SSR markers, but it is very efficient for genes/QTLs mapping since all those markers are derived from expressed sequences. In the early stage of developing molecular markers, we conducted transcriptome sequencing of samples mixing roots, stems, leaves, flowers, and fruits of sponge gourd, covering maximally expressed genes in all sponge gourd tissues, effectively avoiding space-time specificity of gene expression and ensuring the comprehensiveness of EST-SSR markers (Wu et al., 2014). EST-SSRs usually located in non-coding regions of Unigene, where sequences showed relative poor conservatism. Thus, when the PCR-amplified target sequences of 177 pairs of EST-SSR molecular markers were used to build the syntenic relationships with the genomic sequences of cucumber, melon and watermelon, most orthoglogs were obtained. While when Unigene sequences where markers located were used to conduct this syntenic analysis, orthoglogs could be found in almost all sequences. The results of syntenic analysis were quite complicated, indicating that sponge gourd was genetically distinct from cucumber, melon and watermelon, and during the long evolutionary process, incidents such as complex rearrangement inner or out of chromosomes.

As to *Luffa* genus, the flowering time of *L. acutangula* differs by nearly 12 h from *L. cylindrica*, so when the former blooms, the latter has withered away. In addition, pollinators usually get busy at a set period of time. Some pollinators are active in the late afternoon, while some of them are active in the early morning. Therefore, it is hardly possible for pollinators working in the late afternoons to carry pollens from *L. acutangula* to the stigma of *L. cylindrical* blossoming in the early mornings. This could be another reason that their hybirds could not be generated naturally. This is the result of long-term evolutionary selection in nature, which not only is an interesting natural phenomenon, but also hinders the gene flow between them by means of physical isolation, ensuring species identity. At present, the mechanisms of gene control over the flowering time have been explored in depth in model plants; many flowering-control related genes such as *FT, HD1, HD3a, EHd1* have been cloned (Yano et al., 2000; Kojima et al., 2002; Doi et al., 2004; Rubio and Deng, 2007). A circadian clock–controlled flowering pathway in *Arabidopsis thaliana* consists of the genes *GIGANTEA* (*GI*), *CONSTANS* (*CO*), and *FLOWERING LOCUS T* (*FT*). *GI* is a main mediator between the circadian clock and *CO*, which is the master regulator of photoperiodic flowering time control *CO* upregulates the expression of “florigen” *FT*, thereby accelerating time required to flower. (Mizoguchi et al., 2005; Harmer, 2009; Xu et al., 2016) However, the studies have mainly concentrated on the rhythm of plant flowering in one years (flowering date, days from sowing to flowing or the first flowering time of plant), and the rhythm of plant flowering in 1 days (daily flowering time) is rarely studied. As to *Luffa* genus, there is about 12-h difference between the flowering times of *L. acutangula* and *L. cylindrica*, from which we can infer that they may are different in genes controlling the connection between circadian rhythm and flowering time. This provides valuable experimental materials for research concerning the daily flowering rhythm of plant. Recently, Cui et al. (2015) have located 8 QTL related to the flowering time of sponge gourd, among which the interpretable phenotypic variance of QTL ft1.1 reached to 24.61%. In our research, 2 QTL controlling flowering time have been located, in which the *qFT1.2* could explain 22.71% of the PV. Unfortunately, it is hard to make a comparison between the two results because of the SRAP random primer markers used by Cui et al. (2015).

Hybrid sterility was one of the most common form of mechanisms of poszygotic isolation between species or subspecies, which led to genetic differentiation and speciation, and also contributed to the maintenance of species identity (Orr and Presgraves, 2000; Long et al., 2008). The Dobzhansky-Muller model of hybrid incompatibility indicated that hybrid sterility and inviability were caused by negative genetic interactions existing between loci that accumulated substitutions in diverging lineages (Dobzhansky, 1936; Doi et al., 2004; Moyle and Nakazato, 2010). A few interactive genes caused segregation distortion and hybrid incompatibility based on researches in animal models such as mice and Drosophila (Civetta and Gaudreau, 2015; Davies et al., 2016). According to studies in plants, hybrid sterility is one of the major forms of postzygotic reproductive isolation, and there are a few genes which are consistent with Dobzhansk-Muller model relating to reproductive isolation (Bomblies and Weigel, 2007; Bikard et al., 2009). Correlational studies in Arabidopsis and rice were conducted in depth, while seldom found in sponge gourd now. Our research identified 4 QTL relating to hybrid male sterility, and the effective value of two of them were more than 10%, which established the foundation for cloning relative hybrid sterility genes and exploring the mechanisms of hybrid sterility of sponge gourd.

The different flowering times and hybrid male sterility form two obstacles to the interspecies cross of *L. acutangula* and *L. cylindrica*, and beyond that, there are maybe other barriers such as female sterility and non-budding of hybrid seeds, which needs our further study in order to reveal the mechanism of interspecific differentiation of sponge gourd, contributing to the change of reproductive isolation in breeding practices and use of heterosis possessed by interspecific hybrid.

## Author contributions

HW conceived, organized and planned the research, data analysis, and drafted the manuscript. HG, XH, and SL contributed to linkage map construction and QTL analyses. ML, JC conducted the EST-SSR polymorphism tests. CZ, WH grew the plant materials and conducted trait measurements. TY conducted colinearity analysis between the sequences of EST-SSR markers and cucumber, melon and watermelon genome, respectively. JL participated in designing the experiments, organized and reviewed the manuscript. All authors read and approved the final manuscript.

### Conflict of interest statement

The authors declare that the research was conducted in the absence of any commercial or financial relationships that could be construed as a potential conflict of interest. The reviewer ZB and handling Editor declared their shared affiliation, and the handling Editor states that the process nevertheless met the standards of a fair and objective review.

## Supplementary material

Supplementary Material 1 **Characterization of 405 EST-SSR primers developed in sponge gourd**.

Supplementary Material 2 **The result of colinearity analysis between the sequences of EST-SSR markers and cucumber genome**.

Supplementary Material 3 **The result of colinearity analysis between the sequences of EST-SSR markers and melon genome**.

Supplementary Material 4 **The result of colinearity analysis between the sequences of EST-SSR markers and watermelon genome**.
